# Novel combination closure of an artificial ulcer after gastric endoscopic submucosal dissection using a double-arm-bar suturing system and an anchor prong clip

**DOI:** 10.1055/a-2603-7290

**Published:** 2025-06-18

**Authors:** Yasunori Yamamoto, Hirohito Mori, Masaya Okada, Teruki Miyake, Eiji Takeshita, Yoshiou Ikeda, Yoichi Hiasa

**Affiliations:** 1 Endoscopy Center, Ehime University Hospital, Toon, Ehime, Japan; 2 Department of Advanced and Innovative Endoscopy, Ehime University Graduate School of Medicine, Toon, Ehime, Japan; 3 38050 Department of Gastroenterology and Metabology, Ehime University Graduate School of Medicine, Toon, Ehime, Japan; 4 Department of Inflammatory Bowel Diseases and Therapeutics, Ehime University Graduate School of Medicine, Toon, Ehime, Japan

Mucosal defect closure after endoscopic submucosal dissection (ESD) is expected to reduce delayed bleeding, especially in patients taking antithrombotic drugs 1 . However, suturing is difficult due to the thick gastric mucosa and muscle layer 2 . Although the double-arm bar suturing system ( Fig. 1 **a–c** , Zeosuture M; Zeon Medical Co.) allows accurate and strong endoscopic suture, it requires many sutures to reduce submucosal dead space, resulting in longer procedure times and higher medical costs 3 4 . Recent studies have reported that a novel anchor prong clip with strong grasping force ( Fig. 1 **d** , MANTIS Clip; Boston Scientific) is useful for mucosal defect closure to reduce submucosal dead space after ESD 5 . In this case report, we successfully achieved a more efficient and secure closure of a mucosal defect after gastric ESD with minimal submucosal dead space by combining the double-arm bar suturing system and the anchor prong clip ( Video 1 ).

**Fig. 1:**
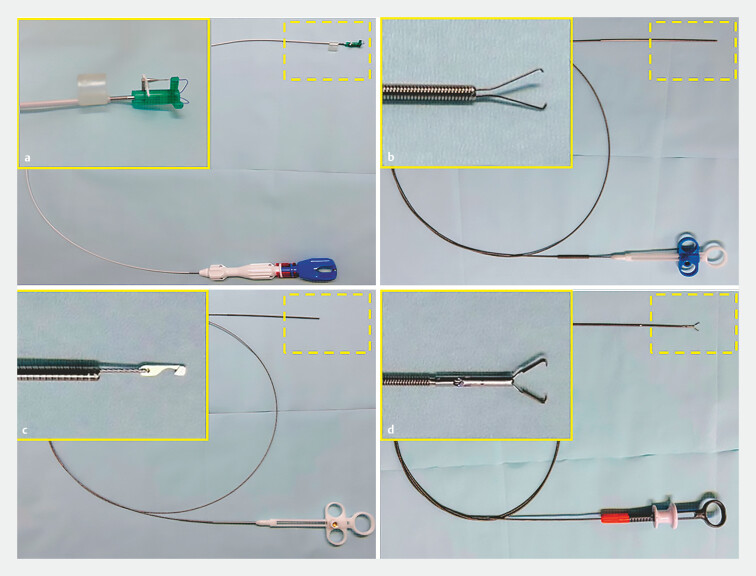
Endoscopic suturing devices and a novel clip. **a** Zeosuture M (Zeon Medical Co.). **b** Zeotieupper S (Zeon Medical Co.). **c** Hookcutter MI (Zeon Medical Co.). **d** MANTIS Clip with a sharp anchor that can be rotated and regripped (Boston Scientific).

Successful closure of a mucosal defect after gastric endoscopic submucosal dissection with a novel combination closure method using the double-arm-bar suturing system and an anchor prong clip.Video 1

A 70-year old man taking dual antiplatelet therapy (DAPT) underwent ESD for a 30 mm lesion in the greater curvature of the lower gastric body. DAPT was not discontinued during treatment. Mucosal resected edge suturing was performed at three sites using endoscopic suturing with Zeosuture M, Zeotieupper S, and Hook Cutter MI. Next, while suctioning air, the muscular layer was approximated and closed with nine MANTIS clips ( Fig. 2 ). The total procedure time for closure was 42 minutes. Endoscopic examinations on postoperative days 1, 7 and 30 confirmed sustained closure ( Fig. 3 ). No delayed bleeding or delayed perforation occurred.

**Fig. 2:**
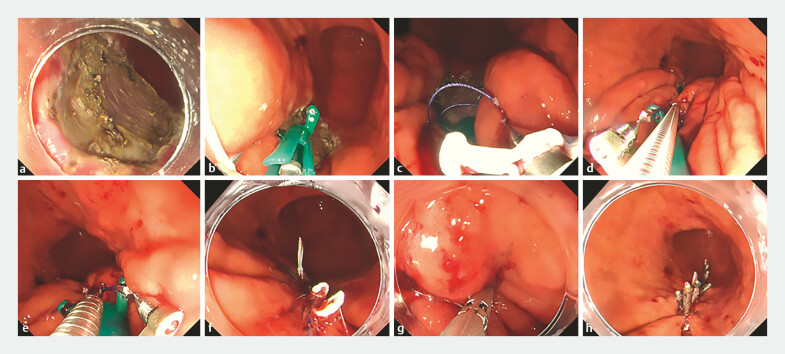
**a** Mucosal defect in the greater curvature of the gastric body after endoscopic submucosal dissection. **b** The left side of the forearm was placed between the mucosal defect and the mucosal valve and the posterior arm with the puncture needle was advanced so that the needle penetrated the gastric mucosa. **c** The right side of the forearm was then placed, and the mucosa was penetrated with a puncture needle with a suture. **d** Both sides of the gastric mucosa were tied together at the mucosal defect and tension was applied to the suture with a Zeotieapper S. **e** After the suture was fixed, the remained thread was cut with the Hookcutter MI. **f** The three sutures were completed. **g** The muscle layer was pulled with suction and closed using new anchor prong clips using strong grasping force. **h** The sutures and clips were placed and the mucosal defect was completely closed with minimal submucosal dead space.

**Fig. 3:**
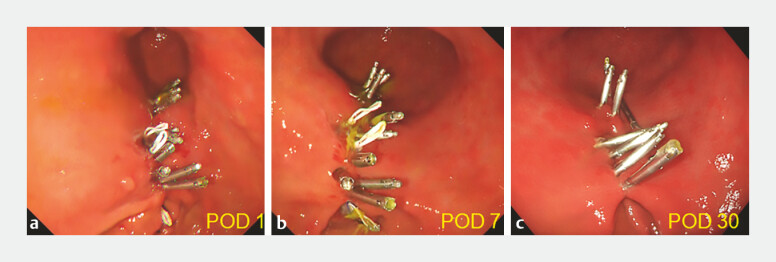
Successful complete closure of the mucosal defect on postoperative days 1 and 7. Day 1 after gastric ESD. Day 7 after gastric ESD. Day 30 after gastric ESD. Abbreviations: ESD, endoscopic submucosal dissection; POD; post operative day.

This method made it possible to close the post-ESD ulcer defect more simply and tightly with a combination of precise mucosal suturing using Zeosuture M and minimizing submucosal dead space using MANTIS clips. This suturing technique may be useful in preventing adverse events after ESD in high-bleeding risk patients.

Endoscopy_UCTN_Code_TTT_1AO_2AO

## Notes

Endoscopy E-Videos https://eref.thieme.de/e-videos
*E-Videos* is an open access online section of the journal *Endoscopy* , reporting on interesting cases and new techniques in gastroenterological endoscopy. All papers include a high-quality video and are published with a Creative Commons CC-BY license. Endoscopy E-Videos qualify for HINARI discounts and waivers and eligibility is automatically checked during the submission process. We grant 100% waivers to articles whose corresponding authors are based in Group A countries and 50% waivers to those who are based in Group B countries as classified by Research4Life (see: https://www.research4life.org/access/eligibility/ ). This section has its own submission website at https://mc.manuscriptcentral.com/e-videos .

## References

[1] Kobara H Tada N Fujihara S Clinical and technical outcomes of endoscopic closure of postendoscopic submucosal dissection defects: Literature review over one decade Dig Endosc 2023 35 216 231 10.1111/den.14397 35778927

[2] Choi KD Jung HY Lee GH Application of metal hemoclips for closure of endoscopic mucosal resection-induced ulcers of the stomach to prevent delayed bleeding Surg Endosc 2008 22 1882 1886 10.1007/s00464-008-9743-0 18270775

[3] Minato Y Mori H Ito F Endoscopic suturing using a new device to prevent adverse events after endoscopic submucosal dissection: Double-arm-bar Suturing System Dig Endosc 2022 34 e9 e11 10.1111/den.14158 34668242

[4] Mori H Kobara H Kazi R Balloon-armed mechanical counter traction and double-armed bar suturing systems for pure endoscopic full-thickness resection Gastroenterology 2014 147 278 2800 24973723 10.1053/j.gastro.2014.06.030

[5] Inada T Sumida Y Homma H Novel clip method for endoscopic submucosal dissection defect closure reducing submucosal dead space in antithrombotic gastric patients Endoscopy 2024 56 01 E45 E46 10.1055/a-2223-4475 38232769 PMC10794086

